# Protective Effects of the Soluble Epoxide Hydrolase Inhibitor 1-Trifluoromethoxyphenyl-3-(1-Propionylpiperidin-4-yl) Urea in a Rat Model of Permanent Middle Cerebral Artery Occlusion

**DOI:** 10.3389/fphar.2020.00182

**Published:** 2020-02-28

**Authors:** Linlei Zhang, Shasha Xu, Xiaoxiao Wu, Farah Mohamed Muse, Jiaou Chen, Yungang Cao, Jueyue Yan, Zicheng Cheng, Xingyang Yi, Zhao Han

**Affiliations:** ^1^ Department of Neurology, The Second Affiliated Hospital and Yuying Children’s Hospital of Wenzhou Medical University, Wenzhou, China; ^2^ Department of General Intensive Care Unit, The Second Affiliated Hospital, College of Medicine, Zhejiang University, Hangzhou, China; ^3^ Department of Neurology, People’s Hospital of Deyang City, Deyang, China

**Keywords:** TPPU, blood brain barrier, cerebral ischemia, apoptosis, tight junction

## Abstract

Acute ischemic stroke is a serious disease that endangers human health. In our efforts to develop an effective therapy, we previously showed that the potent, highly selective inhibitor of soluble epoxide hydrolase called 1-trifuoromethoxyphenyl-3-(1-propionylpiperidin-4-yl) urea (TPPU) protects the brain against focal ischemia in rats. Here we explored the mechanism of TPPU action by assessing whether it could preserve blood-brain barrier integrity and reduce apoptosis in the brain during permanent middle cerebral artery occlusion in male Sprague-Dawley rats. TPPU administration at the onset of stroke and once daily thereafter led to smaller infarct volume and brain edema as well as milder neurological deficits. TPPU significantly inhibited the activity of soluble epoxide hydrolase and matrix metalloproteases 2 and 9, reducing 14,15-DHET levels, while increasing expression of tight junction proteins. TPPU decreased numbers of apoptotic cells by down-regulating the pro-apoptotic proteins BAX and Caspase-3, while up-regulating the anti-apoptotic protein BCL-2. Our results suggest that TPPU can protect the blood-brain barrier and reduce the apoptosis of brain tissue caused by ischemia.

## Introduction

Ischemic stroke, caused by a cerebrovascular blockage, leads to death and long-term disability in many patients around the world (Moskowitz et al., 2010; Benjamin et al., 2018). Ischemic brain cells release inflammatory cytokines that recruit leukocytes and activate microglial cells, leading to breakdown of the blood-brain barrier (BBB) and ischemic brain injury (Wang et al., 2007). While thrombolysis and thrombectomy can be effective treatments (Jauch et al., 2013), they can also exacerbate the cerebral injury, compromise the blood-brain barrier (BBB) and extracellular matrix, and increase the risk of intracerebral hemorrhage (Niego and Medcalf, 2014). Neuroprotectors to mitigate this injury have been reported in animal studies, but most have proven ineffective in clinical trials (O’Collins et al., 2006). Therefore, further efforts are needed to develop agents that can inhibit neuronal cell death and promote brain repair following stroke.

Astrocytes and vascular endothelium in the brain produce endogenous neuroprotectors called epoxyeicosatrienoic acids (EETs), which bind to receptors and thereby activate signal transduction cascades involving cAMP/PKA, PI3K-Akt, and MAPK (Spector and Norris, 2007). EETs can also act through the NF-κB pathway to reduce expression of leukocyte adhesion proteins in endothelial cells (Spector and Norris, 2007). Thus, EETs exert anti-thrombotic, vasodilatory, pro-angiogenic, anti-apoptotic, and anti-oxidant effects (Peng et al., 2002; Iliff et al., 2010; Xu et al., 2011). EETs are rapidly catabolized by soluble epoxide hydrolase (sEH) into less bioactive products such as dihydroxyeicosatrienoic acids (DHETs) (Yu et al., 2000), and the extent of this degradation correlates with deterioration of neurological function, carotid artery stenosis, and plaque instability in patients with cerebral infarction (Yi et al., 2016; Yi et al., 2017).

Therefore, inhibiting sEH has attracted much attention as a potential therapy to protect brain tissue from hypoxic or ischemic damage following stroke (Li et al., 2012). The enzyme is present in vascular endothelial cell layers of cerebral vasculature as well as in neurons in the brain parenchyma, especially in the cerebral cortex (Zhang et al., 2007). Consistent with its potential as a therapeutic target, knocking out the sEH gene in mice increases cerebral blood flow and decreases infarct volume following cerebral middle artery occlusion (MCAO) (Zuloaga et al., 2014), and inhibiting the enzyme can lead to persistent EET signaling that protects against ischemic damage in experimental models (Dorrance et al., 2005; Zhang et al., 2008; Chang et al., 2018). Literature has shown that TPPU lowered the mRNA expression of pro-inflammatory cytokines and promoted reparative cytokines and growth factors. Thus, inhibition of sEH reduced infarction and protected ischemic brain (Tu et al., 2018). Meanwhile TPPU could block activity of sEH and attenuate post-ischemic neuronal hyperexcitation against stroke with TrkB activation (Chang et al., 2018). Other inhibition of sEH such as AUDA enhanced processes of anti-inflammatory and anti-oxidative and modulated microglia polarization after ischemic stroke (Yeh et al., 2019b).

The synthetic compound 1-trifluoromethoxyphenyl-3-(1-propionylpiperidin-4-yl) urea (TPPU) inhibits sEH activity in the central nervous system (Rose et al., 2010), thus increasing levels of EETs (Ulu et al., 2012). In fact, TPPU shows a higher maximum concentration in blood (C_max_) and larger area under the concentration–time curve than adamantyl-containing urea-based inhibitors. In other words, TPPU is absorbed rapidly, absorbed efficiently and eliminated slowly, so it can persist in the blood longer than other hydrolase inhibitors. In this way, TPPU offers greater metabolic stability (Liu et al., 2013). TPPU is more effective at preventing myocardial fibrosis after myocardial infarction and at exerting anti-apoptotic, anti-inflammatory and anti-oxidative effects to protect cardiac and renal tissue in various animal models (Sirish et al., 2013; Tu et al., 2018). TPPU can also inhibit not only excitotoxicity in early stages of cerebral ischemia but also apoptosis in later stages (Dirnagl et al., 1999). These proven results suggest that TPPU may protect against ischemic brain injury in rodents by maintaining the integrity of the BBB and decreasing the number of apoptotic cells.

In the present study, we examined whether TPPU could protect against ischemia-induced BBB damage and apoptosis in the brain. We examined the effects and mechanism of TPPU in rats subjected to permanent MCAO (pMCAO). We focused on the potential effects of TPPU on the expression of three tight junction proteins (ZO-1, occludin, claudin-5) whose down-regulation in cerebral ischemia compromises the BBB (Fernandez-Lopez et al., 2012). We also examined the effects of TPPU on matrix metalloproteases (MMPs) 2 and 9, which are activated during cerebral ischemia and degrade the basal lamina of the BBB, further compromising its integrity (Chen et al., 2016). The resulting permeabilization of the natural barrier leads to brain edema (Hacke et al., 2008). We further found that TPPU decreased the numbers of apoptotic cells by down-regulating the pro-apoptotic proteins BAX and Caspase-3, while up-regulating the anti-apoptotic protein BCL-2. These results show that TPPU can protect the ischemic brain by mitigating BBB disruption and reducing apoptosis caused by ischemia.

## Materials and Methods

### Experimental Reagents and Materials

TPPU, dimethyl sulfoxide (DMSO), 2,3,5-triphenyltetrazolium chloride (TTC), and the *in situ* Cell Death Detection kit were purchased from Sigma (St Louis, MO, USA). Silicon-coated surgical nylon monofilaments were purchased from Guangzhou Jialing Biotechnology (catalog no. L3400; Guangzhou, China). Polyclonal antibodies against sEH and the tight junction proteins Occludin, ZO-1 and Claudin-5 were obtained from Affinity Biosciences (Cincinnati, OH, USA). Antibodies against the apoptosis proteins Caspase-3, BAX, and BCL-2 were obtained from Cell Signaling Technology (Danvers, MA, USA). Antibody against the loading control GAPDH was purchased from Bioworld (MN, USA). Horseradish peroxidase-conjugated secondary anti-rabbit antibody was obtained from EarthOx (San Francisco, USA). TPPU was dissolved in DMSO and stored at 4°C. Before use, it was diluted with physiological saline to reduce the DMSO concentration to 5%.

### Experimental Animals

Healthy adult male Sprague-Dawley rats (n=138, 250–280 g) were purchased from the Wenzhou Medical University Laboratory Animal Center (Wenzhou, China). Only male rats were used because estrogen can mitigate cerebral infarction following MCAO (Khan et al., 2015). All experiments were approved by the Institutional Animal Care and Research Committee of Wenzhou Medical University, and procedures were carried out in accordance with the US National Institutes of Health publication “Guidelines for the Care and Use of Laboratory Animals”.

Rats were fed in a controlled environment (23 ± 2°C, 45–55% relative humidity, 12-h dark/light cycle) and provided with food and water *ad libitum*.

### Permanent MCAO

Of the 138 animals, 102 were subjected to pMCAO using the intraluminal filament technique as described (Zhang et al., 2012). In brief, rats were anesthetized and maintained under anesthesia using, respectively, 3% and 1% isoflurane and an animal anesthesia apparatus (RWD Life Science, Shenzhen, China). A midline incision was made in the neck, and the left common carotid artery and left external carotid artery (ECA) were carefully exposed and isolated from the surrounding connective tissues. The left ECA stump was cut, and a silicon-coated surgical nylon monofilament was gently inserted into the internal carotid artery (ICA) until the mark on the filament reached the ICA-ECA bifurcation. The remaining 36 rats underwent the same procedure but without blockage of the MCA. Then the neck incision of all animals was sutured. All surgical operations were performed under an operating stereomicroscope, and rectal temperature was monitored and maintained at 37°C using a thermostatically controlled heating pad.

### Treatments Following MCAO

The 10 sham rats were injected intraperitoneally 5% DMSO as control. The 50 rats subjected to pMCAO were immediately randomized into five groups and injected intraperitoneally with TPPU (0.5, 1, 1.5, or 2 mg/kg) in 5% DMSO or with 5% DMSO vehicle (all injections were 1 mL). Each group contained 10 animals. The TPPU and DMSO animals were injected once immediately after pMCAO, then killed on day 1. In the initial phase of the study, 60 animals were sacrificed at 24 h after surgery, and brains were analyzed in order to determine the best TPPU concentration. This concentration was then used in subsequent experiments with the remaining 78 animals. Each of the six groups contained 13 animals and were subdivided into “1 day” or “3 days”. Rats killed on day 1 after pMCAO were injected with TPPU (1 mg/kg) or 5% DMSO once immediately after the procedure. Rats killed on day 3 after pMCAO were injected with TPPU or 5% DMSO once a day for 3 days after the procedure. Animals that died during or after the procedure were not analyzed.

### Neurological Dysfunction

At 24 h after surgery, rats were euthanized with 1% pentobarbital sodium (40 mg/kg) and evaluated using the modified neurological severity scores (mNSS) protocol (Komotar et al., 2007), involving motor, sensory, balance, and reflex tests. Scores could range from 0 to 18, with high scores indicating more serious neurological impairment. Assessors were blinded to group allocation.

### Infarct Volume

At 24 h after surgery, rats were sacrificed using 1% pentobarbital sodium (40mg/kg), the entire brain was sectioned into 2-mm slices using a rat brain mold (RWD Life Science, Shenzhen, China), and serial coronal sections were stained with 2% TTC at 37°C for 30 min. Infarct areas in the ipsilateral hemisphere were quantified using Image-Pro Plus 6.0 (Media Cybernetics, Rockville, MD, USA). Relative infarct volume was calculated as previously described (Hou et al., 2009) to minimize edema-induced error: percentage of corrected infarct volume = (volume of total contralesional cerebral hemispheric tissue - non-infarction zone in the ipsilateral hemisphere)/contralesional hemispheric volume x 100%.

### Brain Water Content

The degree of cerebral edema was assessed using wet-to-dry weight ratios. Rats were anesthetized after pMCAO for 24 h. Brains were divided into left and right hemispheres, and the infarct hemispheres were weighed on an electronic balance with an accuracy of 0.01 mg and then dried in an oven at 65°C for 72 h. Dried brain tissues were reweighed to calculate brain water content as: (wet weight–dry weight)/wet weight × 100% (Li et al., 2009).

### Mortality

The mortality rate of an animal group was calculated as the number of dead rats divided by the total number of rats originally allocated to the group (sham group: n = 23; DMSO group: n = 27; TPPU 0.5mg/kg group: n = 12; TPPU 1 mg/kg group: n = 25; TPPU 1.5 mg/kg group: n = 11; TPPU 2 mg/kg group: n = 12).

### Histopathology

At 24 and 72 h after surgery, rats were sacrificed using 1% pentobarbital sodium, the heart was exposed and perfused with 0.9% sodium chloride, then fixed with 50 mL of 4% paraformaldehyde dissolved in phosphate-buffered saline (PBS). Brains were removed and immersed in the same fixative for 24 h to make paraffin blocks. Brain tissues were sectioned into 5-µm thick slices, cortical sections were stained with hematoxylin-eosin as described (Zhang Z. et al., 2018), and stained slices were observed in a blinded fashion by a pathologist under a microscope (Olympus Optical, Tokyo, Japan). These pictures were used to verify model success and find the ischemic penumbra of the cortex.

### Activity of Brain sEH

Brain cortex tissue was homogenized in 4 volumes of cold 20 mmol/L Tris-HCl (pH 7.4) containing 0.32 mol/L sucrose and 1 mmol/L EDTA. The homogenate was centrifuged at 1000 g for 10 min, then the supernatant was transferred to a clean tube and centrifuged at 10,000 *g* for 20 min. The supernatant was incubated with 14,15-EET for 1 h, and levels of 14,15-DHET were assayed using a commercial ELISA (Detroit R&D, Detroit, MI, USA) based on absorbance at 450 nm (UV-6100S; Meipada Instrument Co., Shanghai, China).

### Apoptosis

Numbers of apoptotic cells in brain cortical sections were assessed using the *in situ* Cell Death Detection Kit as described (Zhang H. et al., 2018). Parafin-embedded sections were dewaxed, incubated with 20 µg/ml proteinase K, washed three times in PBS, and incubated with TdT/dUTP FITC labeling mixture for 1 h at room temperature. Sections were examined under a microscope at ×100 magnifcation.

### Matrix Metalloprotease Activity

The activity of matrix metalloproteases (MMPs) 2 and 9 in brain cortical ischemic penumbra were assayed using gelatin zymography as described (Fujimura et al., 1999). Equal volumes of protein extracts were fractionated on 10% SDS polyacrylamide gels containing 0.1% gelatin, the gels were rinsed three times in 2.5% Triton X-100 (pH 7.4) for 40 min, then the gels were incubated in zymography buffer [50 mM Tris–HCl (pH 7.6), 50 mM NaCl, 5 mM CaCl2] for 42 h at 37°C. Gels were stained with 0.05% Coomassie brilliant blue for 3 h, then destained in 30% methanol and 10% glacial acetic acid. MMP2 and MMP9 bands were analyzed using Image J software (National Institutes of Health, Bethesda, MD, USA).

### Levels of Apoptosis-Related Proteins and Tight Junction Proteins

Tissue samples of brain ischemic penumbra were lysed in buffer (Solarbio Technology, Beijing, China) containing the protease inhibitor phenylmethylsulfonyl fluoride (Beyotime Biotechnology, Shanghai, China), the lysate was centrifuged at 12,000 *g* for 20 min at 4°C, and protein concentration in the supernatant was assayed using the BCA Assay Kit (Beyotime Biotechnology) according to the manufacturer’s instructions. The clarified lysates were boiled and stored at -20°C. Equal amounts of proteins (30 μg) were fractionated on 10% SDS-PAGE, then transferred to 0.45-μm or 0.20-μm polyvinylidene fluoride (PVDF) membranes. Membranes were analyzed by Western blotting as previously described (Ashwal et al., 1999): they were blocked for 1.5 h in 5% nonfat milk solution containing TBS with 0.05% Tween 20, rinsed, and incubated overnight at 4°C with primary antibodies against sEH (diluted 1:500), BAX (1:1,000), BCL-2 (1:1,000), Caspase-3 (1:1,000), Occludin (1:1,000), Claudin-5 (1:1,000), ZO-1 (1:1,000) or GAPDH (1:5,000). Membranes were rinsed three times, incubated with horseradish peroxidase-conjugated secondary antibody (1:3,000) for 1.5 h at room temperature, washed three times, then finally soaked in enhanced chemiluminescent (ECL) substrate (Pierce Chemical, IL, USA). Protein bands were visualized using the bio-imaging system (Upland, CA, USA), and band intensities were quantified using AlphaEaseFC 4.0 (Alpha Innotech, San Leandro, California, USA).

### Statistical Analysis

Data were analyzed using SPSS 17.0 (IBM, Chicago, IL, USA) and reported as the mean ± SEM. Statistical differences between two groups were assessed using Student’s *t* test, and differences among more than two groups were examined by one-way ANOVA. Inter-group differences in mortality rate were assessed using the chi-squared test. Statistical significance was defined as p < 0.05.

## Results

### TPPU Decreased Cerebral Infarct Volume and Brain Edema and Mitigated Neurological Impairment Induced by pMCAO

**Figure 1:**
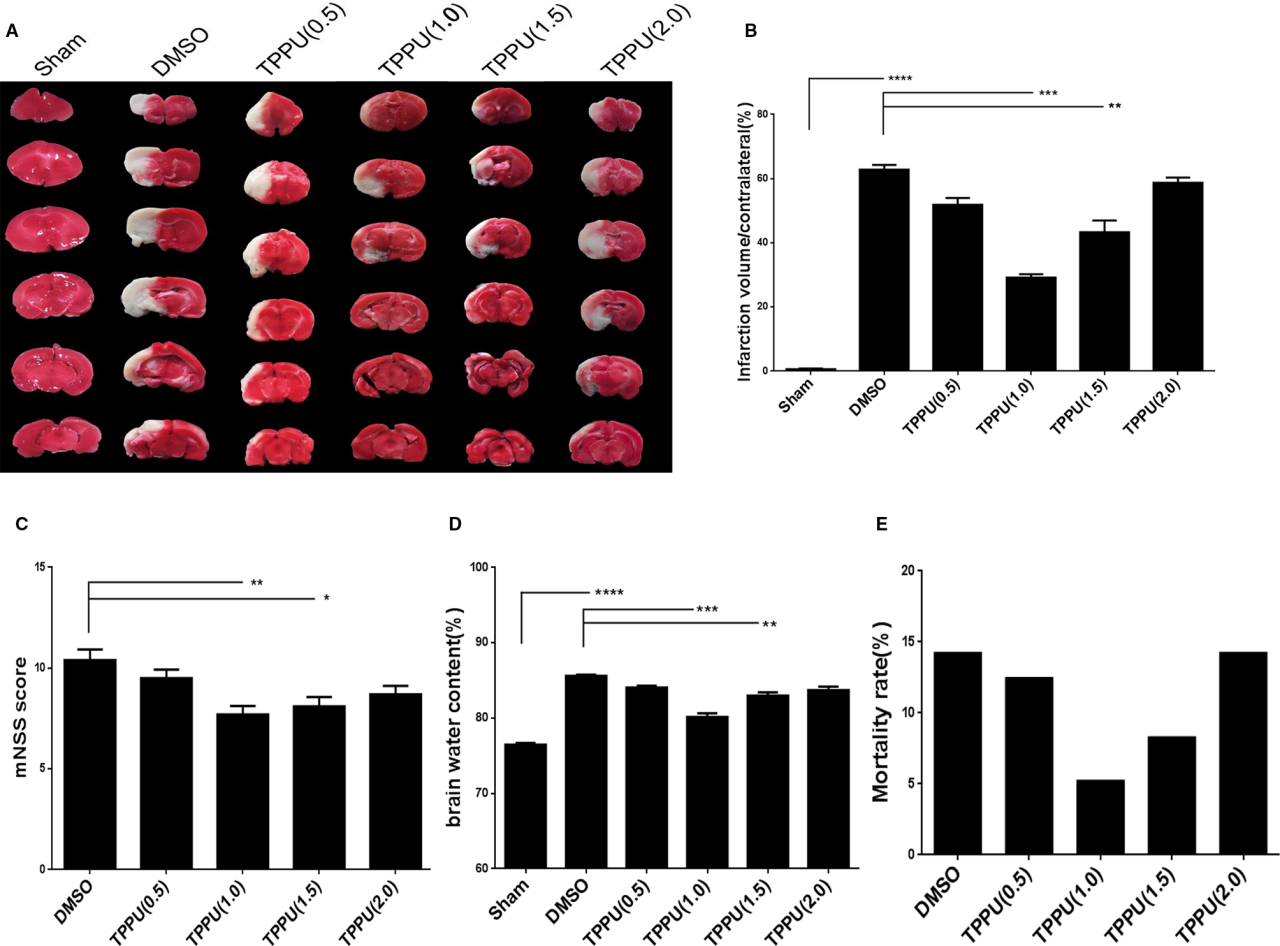
1-trifluoromethoxyphenyl-3-(1-propionylpiperidin-4-yl) urea (TPPU) decreased infarct volume and brain edema, mitigated neurological impairment and reduced mortality during permanent cerebral middle artery occlusion (pMCAO). **(A)** Brains were removed from animals at 3 days after surgery, sectioned, and stained with 2,3,5-triphenyltetrazolium chloride (TTC). **(B)** Quantitation of infarct volume (n = 6). **(C)** Neurological function based on modified neurological severity scores (mNSS) (n = 10). **(D)** Brain edema content in the ischemic hemisphere (n = 4). **(E)** Death of animals within 1 days after surgery (n > = 11). Data are the mean ± SEM. *p < 0.05, **p < 0.01, ***p < 0.001, ****p < 0.0001.

Rats were subjected to pMCAO, immediately followed by injection of TPPU at 0, 0.5, 1, 1.5, or 2 mg/kg. As expected, pMCAO led to a large infarct volume in the ipsilateral hemisphere not seen in the sham-operated animals, and TPPU reduced the infarct volume to varying degrees (**Figure 1A**), with a TPPU dose of 1 mg/kg showing the greatest protective effects, based on histology (**Figure 1B**) and mNSS score (**Figure 1C**).

Similarly, TPPU partially reversed the MCAO-induced increase in brain water content, with the dose of 1 mg/kg showing the greatest effect (**Figure 1D**). TPPU dose did not significantly affect mortality (**Figure 1E**). These results suggest that TPPU can be protective against ischemic brain injury.

### TPPU Mitigated Cell Shrinkage and Denaturation Induced by pMCAO

**Figure 2:**
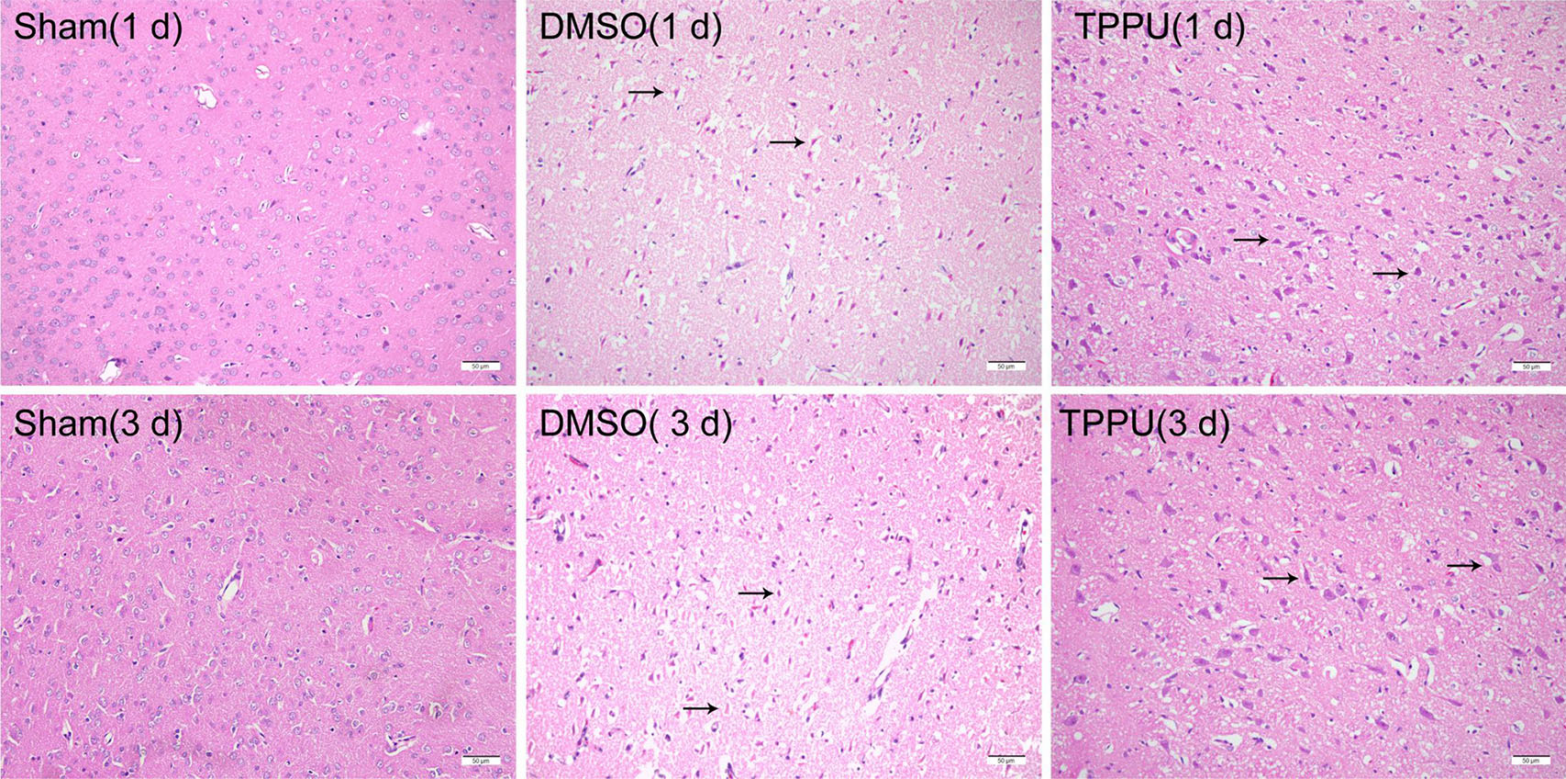
1-trifluoromethoxyphenyl-3-(1-propionylpiperidin-4-yl) urea (TPPU) alleviated cell shrinkage and injury induced by permanent cerebral middle artery occlusion (pMCAO). Brains were removed from animals at 1 or 3 days after surgery, and the cortex was sectioned and stained with hematoxylin-eosin (n = 3). Arrowheads indicate denatured cells and enlarged extracellular spaces in cortical tissue. Scale bar, 50 μm.

After hematoxylin-eosin staining, the cortical area in sham-operated animals showed an intact, regular arrangement with no visible infarcts (**Figure 2**). Permanent MCAO severely damaged and killed cells, leaving vacuolated interspaces. TPPU mitigated these effects at 1 day and 3 days similarly.

### TPPU Reduced sEH Expression and Reinforced BBB Integrity Against pMCAO

**Figure 3:**
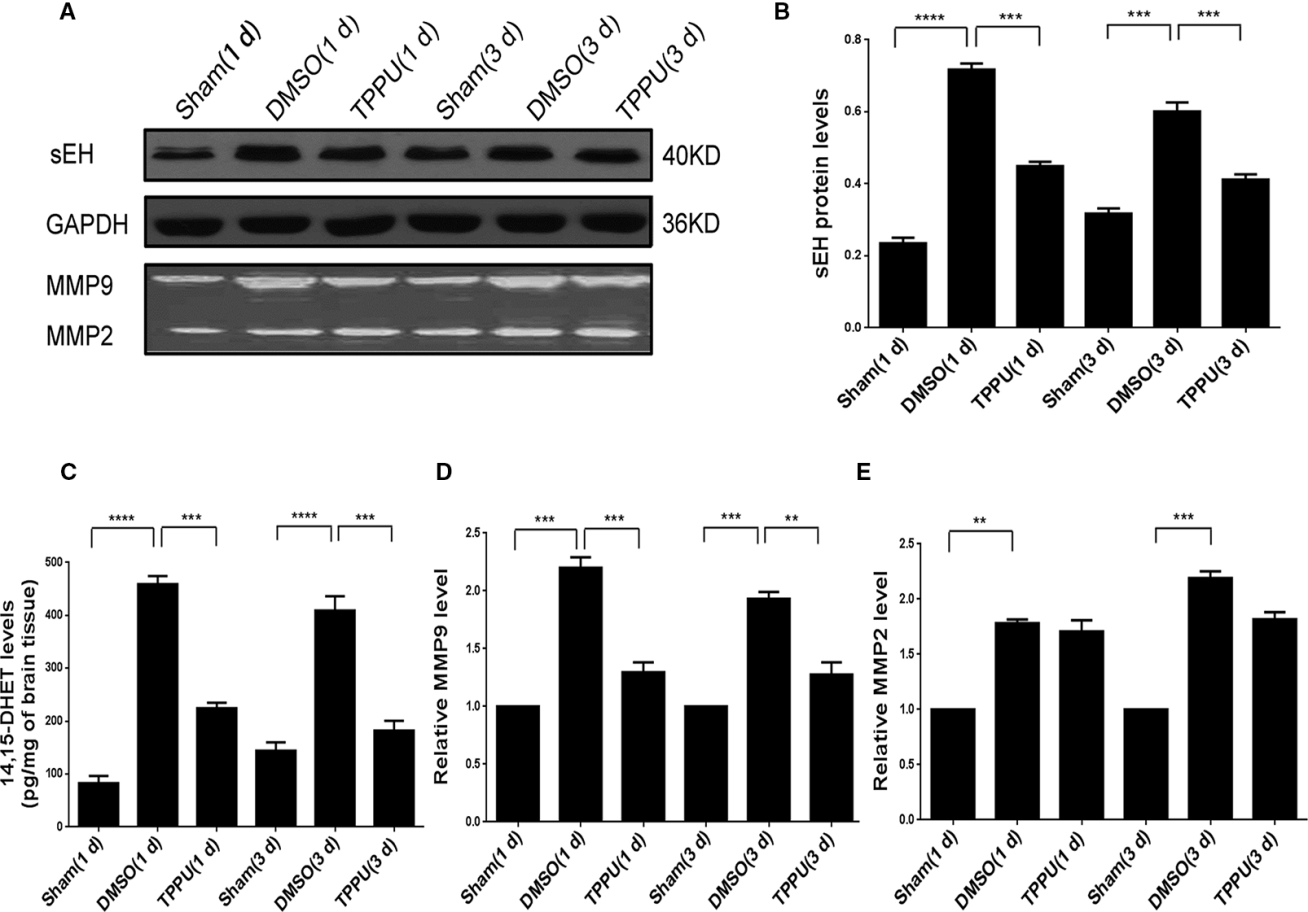
1-trifluoromethoxyphenyl-3-(1-propionylpiperidin-4-yl) urea (TPPU) down-regulated soluble epoxide hydrolase and inhibited matrix metalloprotease activity in the penumbra area during permanent cerebral middle artery occlusion (pMCAO). **(A)** Western blots showing levels of soluble epoxide hydrolase and GAPDH as a loading control (*top and middle panels*), and gel zymography showing gelatinase activity of MMP9 and MMP2 (*bottom panels*). **(B)** Quantitation of levels of soluble epoxide hydrolase relative to levels of GAPDH (n = 5). **(C)** Quantitation of 14,15-DHET levels in brain tissue (in pg/mg, n = 4). **(D)** Quantitation of relative levels of MMP9 (n = 4). **(E)** Quantitation of relative levels of MMP2(n = 4). Data are the mean ± SEM. **p < 0.01, ***p < 0.001, ****p < 0.0001.

Expression of sEH is normally widely distributed in neuronal cell bodies and processes (Zhao et al., 2012). Levels of sEH increased in the ischemic penumbra in the presence of pMCAO, which TPPU treatment at 1 mg/kg partially reversed indirectly by reducing inflammation (**Figures 3A, B**). These changes in sEH protein levels were mirrored by changes in sEH activity based on ELISA of 14,15-DHET (**Figure 3C**). Similarly, pMCAO up-regulated activity of MMP2 and MMP9 (**Figures 3D, E**) based on a gelatinase assay (**Figure 3A**, *upper panel*), and TPPU partially reversed these changes. These results suggest that the protective effects of TPPU against ischemic injury may involve the down-regulation of soluble epoxide hydrolase and inhibition of MMP2 and MMP9 activity.

### TPPU Up-Regulated Tight Junction Proteins, Which May Preserve BBB Integrity Against pMCAO

**Figure 4:**
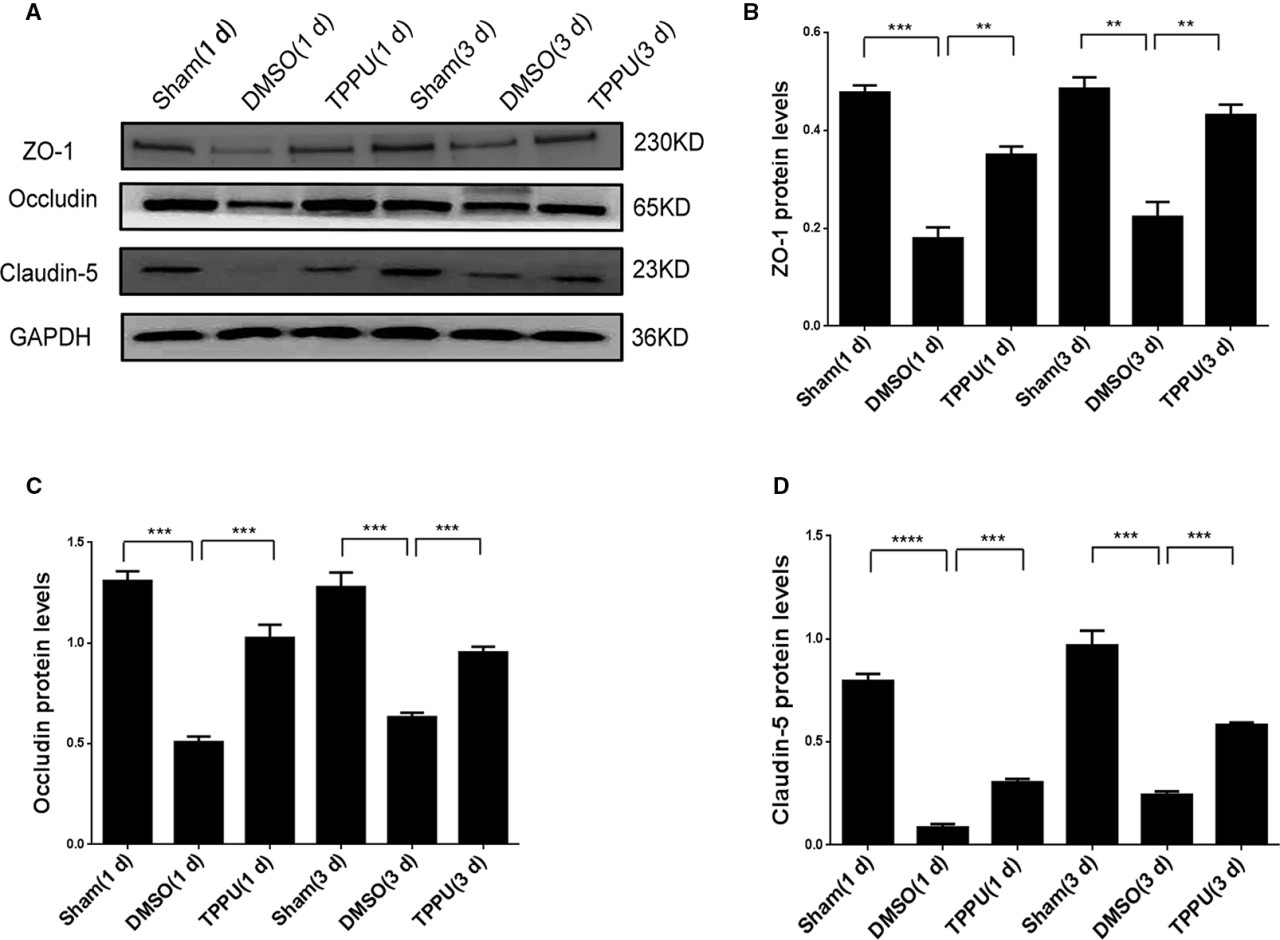
1-trifluoromethoxyphenyl-3-(1-propionylpiperidin-4-yl) urea (TPPU) recovered expression of tight junction proteins down-regulated by permanent cerebral middle artery occlusion (pMCAO). **(A)** Brains were removed from animals at 1 or 3 days after surgery, and cortical tissue was homogenized and assayed by Western blot against Occludin, ZO-1, and Claudin-5. **(B)** Quantitation of relative levels of ZO-1 (n = 5). **(C)** Quantitation of Occludin levels (n = 5). **(D)** Quantitation of relative levels of Claudin-5 (n = 5). Data are the mean ± SEM. **p < 0.01, ***p < 0.001, ****p < 0.0001.

As expected, pMCAO significantly reduced expression of the tight junction proteins ZO-1, Occludin and Claudin-5 in peri-ischemic cortical tissue (**Figure 4**), and this down-regulation compromises BBB integrity (Brown and Davis, 2002). TPPU not covalently bound to sEH rescued the expression of all three proteins, probably by increasing endogenous levels of EETs (**Figures 4B–D**), which may help preserve BBB integrity.

### TPPU Mitigated Cell Apoptosis Induced by pMCAO

**Figure 5:**
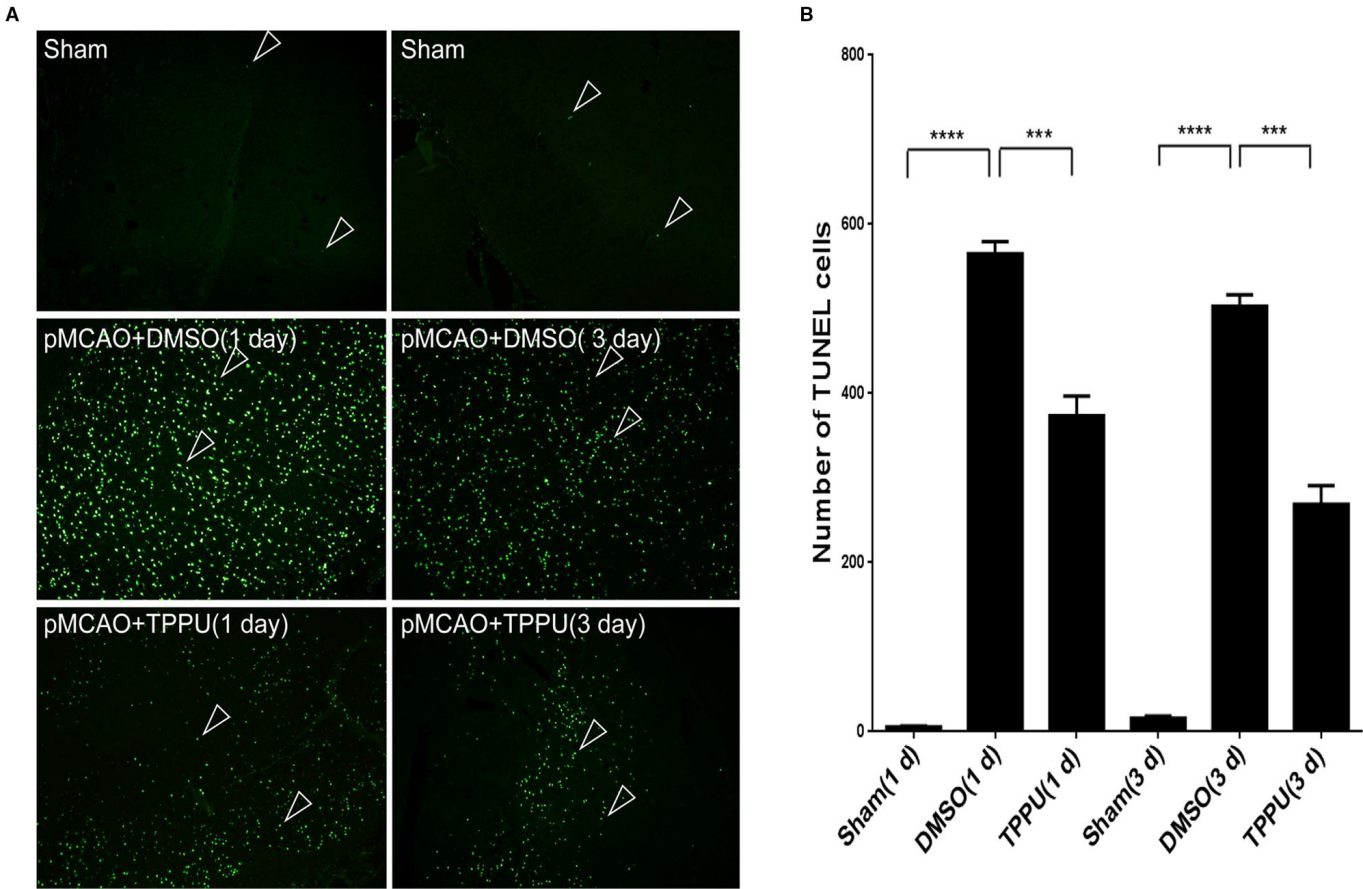
1-trifluoromethoxyphenyl-3-(1-propionylpiperidin-4-yl) urea (TPPU) reduced apoptosis in the ischemic penumbra during permanent cerebral middle artery occlusion (MCAO). **(A)** Brains were removed from rats at 1 or 3 days after surgery, sectioned, and TUNEL-stained. Arrowheads indicate apoptotic cells in cortical tissue. Scale bar, 50 μm. **(B)** Quantitation of TUNEL-positive cells (n = 4). Data are the mean ± SEM.***p < 0.001, ****p < 0.0001.

Permanent MCAO led to a large number of apoptotic cells in the penumbra, which was not observed in sham-operated animals (**Figure 5A**). TPPU partially reversed this induced apoptosis (**Figure 5B**).

### TPPU Inhibited Apoptosis by Up-Regulating BCL-2 and Down-Regulating BAX and Caspase-3

**Figure 6:**
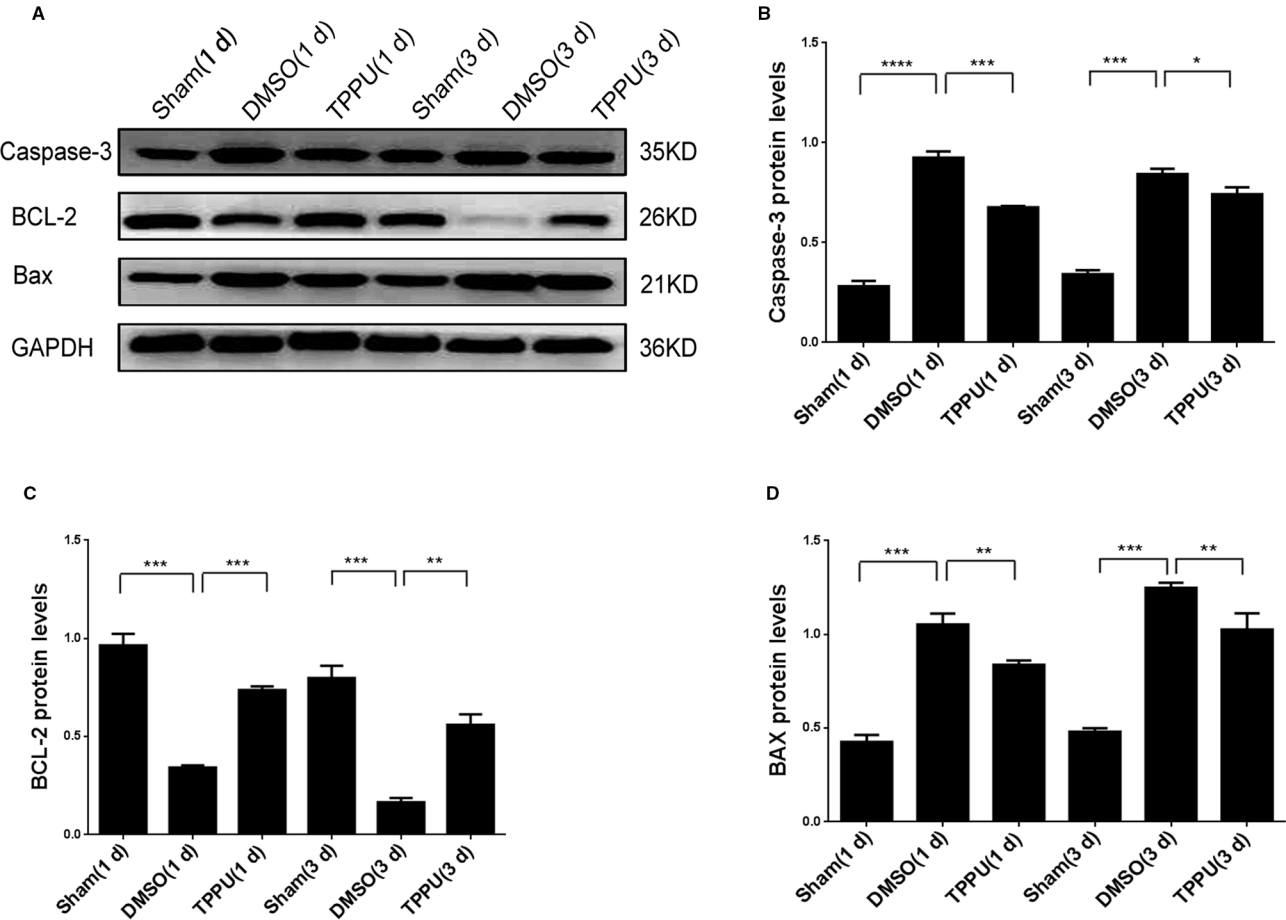
1-trifluoromethoxyphenyl-3-(1-propionylpiperidin-4-yl) urea (TPPU) up-regulated BCL-2 and down-regulated caspase-3 and BAX. **(A)** Brains were removed from animals at 1 or 3 days after surgery, and cortical tissue was homogenized and assayed by Western blot against Caspase-3, BCL-2, and BAX. **(B)** Quantitation of relative levels of Caspase-3 (n = 5). **(C)** Quantitation of relative levels of BCL-2 (n = 5). **(D)** Quantitation of relative levels of BAX (n = 5). Data are the mean ± SEM. *p < 0.05, **p < 0.01, ***p < 0.001, ****p < 0.0001.

Permanent MCAO up-regulated Caspase-3 and BAX and down-regulated BCL-2 (**Figure 6**), consistent with the pro-apoptotic effects of the first two proteins and the anti-apoptotic effects of the last (Chiou et al., 2006; Yu et al., 2011). TPPU partially reversed these MCAO-induced changes. Thus, the neuroprotector may exert its anti-apoptotic effects by altering the balance of apoptosis proteins in brain tissue.

## Discussion

Stroke, which is ischemic in 80% of cases, is the third leading cause of death and long-term disability in developing countries, and it places an enormous economic burden on families (Abilleira et al., 2011; Towfighi and Saver, 2011). No effective therapies for ischemic stroke are currently available (Han et al., 2012). Using a rat model of permanent MCAO, we provide evidence that TPPU, an inhibitor of soluble epoxide hydrolase, can mitigate ischemic damage related to apoptosis and BBB permeabilization.

Our data suggest that these effects of TPPU involve not only down-regulation of the hydrolase and 14,15 DHET levels but also inhibition of MMPs, rescue of tight junction protein expression, and readjustment of at least three apoptosis-related proteins (BCL-2, BAX, and Caspase-3) to reduce the level of apoptosis in the penumbra. The decrease in sEH and 14,15 DHET levels should lead to accumulation of protective EETs because TPPU is a noncovalent sEH inhibitor that binds to the enzyme in a kinetically reversible process (Liu et al., 2013). Our findings raise the possibility that inhibiting this hydrolase as adjunct therapy with thrombolysis and thrombectomy may benefit patients.

TPPU, first mentioned in 2010 (Rose et al., 2010), was synthesized as one of several potential inhibitors in which the adamantyl group was replaced by a phenyl substitution (Liu et al., 2013). It presents several advantages over other sEH inhibitors. For example, 12-(3-adamantan-1-yl-ureido) dodecanoic acid and trans-4-[4-(3-adamantan-1-yl-ureido)-cyclohexyloxy]-bensoic acid can protect cerebral, cardiac and renal tissue from ischemic injury (Dorrance et al., 2005; Iliff and Alkayed, 2009; Roche et al., 2015; Darwesh et al., 2019), but they must be administered multiple times at quite high doses because they are poorly absorbed from the gastrointestinal tract, and they are rapidly metabolized and excreted because the flexible alkyl chain oxidizes easily (Ulu et al., 2012). TPPU can penetrate the intact rat BBB, as confirmed by assays of 14,15-DHET levels, and it can inhibit sEH activity within the brain. In fact, it exerts stronger anti-inflammatory, anti-oxidant, and cardioprotective effects than other hydrolase inhibitors in various animal models (Liu et al., 2013).

While TPPU in our experiments inhibited sEH activity in ischemic tissues in a dose-dependent manner from 0.5 to 1 mg/kg, the highest dose of 2 mg/kg did not work as well as a dose of 1 mg/kg. These results are consistent with a report that the EET : DHET ratio can be lower after 3 mg/kg TPPU than 1 mg/kg TPPU (Liu et al., 2013). We speculated that this may be related to the pharmacokinetic properties of TPPU. Indeed, TPPU reflects a quick and strong absorption and a slow elimination. It persists longer in the plasma due to its metabolic stability (Ostermann et al., 2015): excessive TPPU may precipitate out of the blood due to its limited water solubility, decreasing the absorption rate. In fact, *in vitro* studies have shown that TPPU at 0.1 mg/kg can lead to concentrations higher than the IC_50_ values (Liu et al., 2013). The toxicity of TPPU has not been reported in the literature, which suggests that TPPU is safe at low concentrations. It is very suitable for clinical treatment because high efficiency at low frequency and compliance is high for the patient.

We performed all assessments at 1 and 3 days after initiation of MCAO and did not notice any significant differences. This may reflect the small number of animals and short timeframe of our experiments. We did observe a non-significant tendency for ischemic symptoms to be milder on day 3 than on day 1, based on histopathology, apoptosis, and neurological defects. This may seem inconsistent with the idea that ischemic symptoms gradually worsen until reaching a peak (Tu et al., 2018). However, our results are similar to those obtained with a rat model of MCAO in different days after AUDA treatment (Yeh et al., 2019a). An apparent mitigation of ischemic symptoms over time may reflect gradually decreasing expression of proinflammatory MMP9. We also cannot exclude mortality bias in our experiments: it may be that rats that experienced milder effects from pMCAO tended to recover, whereas severely affected animals died. Under our experimental conditions, 16 of the 154 rats died within 3 days, suggesting that the surgery had less severe effects, such that most animals improved rather than worsened during the 3-day follow-up.

The BBB is the core structure of the neurovascular unit (NVU), which ensures an appropriate microenvironment for normal neuronal function (Nizari et al., 2019). By contributing to tight junctions, the transmembrane proteins Occludin and Claudin-5 as well as the membrane-associated protein ZO-1 in vascular endothelial cells can help maintain or restore BBB integrity (Srivastava et al., 2013), yet they are down-regulated after ischemic stroke. This may be related to sEH activation and a corresponding decrease in EETs. After stroke, neuroinflammation rapidly activates microglia and promotes the infiltration of circulating inflammatory cells (Jin et al., 2010). In the acute phase, high expression of MMPs and reactive oxygen species promotes ischemic injury by disrupting the BBB and triggering neuronal death (Jin et al., 2010). Excessive levels of reactive oxygen species injure and kill cells, trigger inflammation, and induce MMP9 expression, leading to BBB breakdown (Chuang et al., 2015). TPPU rescued expression of tight junction proteins down-regulated by pMCAO, presumably allowing them to reduce ischemia-induced BBB permeability by increasing levels of EETs, which exert anti-thrombotic, vasodilatory, pro-angiogenic, anti-apoptotic, and anti-oxidant effects (Imig, 2012). Studies have shown that TPPU increased the density of blood vessels in the ischemic brain but not altered blood flow during the ischemic period (Iliff and Alkayed, 2009; Tu et al., 2018).

Cerebral ischemia stroke gives rise to neuronal death, at least in part by activating apoptosis. Ischemia causes the production of reactive oxygen species that injure mitochondria, activating the pro-apoptotic protein BAX, which induces mitochondrial release of cytochrome C and activation of Caspase-9 (Qi et al., 2014). Subsequently, the pro-apoptotic Caspase-3 activates and triggers proteolytic degradation and ultimately cell death. This cascade can be inhibited by anti-apoptotic BCL-2 family proteins (Li et al., 2010). Our TUNEL assays and Western blots against BAX, caspase-3, and BCL-2 point to the same conclusion: TPPU reverses MCAO-induced apoptosis in the brain. The literature has revealed that AUDA modulated gene expression of mediators involved in the regulation of apoptosis in neural tissue of rats with a shift in the balance, producing possible ischemic tolerance (Simpkins et al., 2009). While overexpression of SEH increased cell death. These datas were also in agreement with previous reports that protecting endothelial cells and neurons from pro-inflammatory mediators induced apoptosis by MAPK or PI3/Akt or BDNF signaling pathway in ischemic brain tissue (Hung et al., 2017; Hao et al., 2018; Park et al., 2019). We speculate that TPPU may exert this effect by reducing ischemia-induced mitochondrial damage and therefore mitochondria-mediated apoptotic signaling (Rekuviene et al., 2017). These results mean that TPPU acts *via* at least two mechanisms to protect brain tissue following stroke: preserving BBB integrity and reducing apoptosis. Future work should seek to clarify how TPPU exerts these effects, including whether EETs or their bioactive metabolites are the mediators. Future studies should also seek to examine whether and how TPPU reverses apoptosis in astrocytes, neurons, or both cell types.

## Conclusions

In summary, our results suggest that inhibiting sEH with TPPU can mitigate ischemia-induced apoptosis in brain tissue and BBB damage by increasing EETs levels. Further work in animals and ultimately humans is needed to identify the mechanisms of TPPU action and its downstream targets. This drug may turn out to be effective against ischemic stroke.

## Data Availability Statement

The datasets generated for this study are available on request to the corresponding authors.

## Ethics Statement

All experiments were approved by the Institutional Animal Care and Research Committee of Wenzhou Medical University, and procedures were carried out in accordance with the US National Institutes of Health Guidelines for the Care and Use of Laboratory Animals.

## Author Contributions

All authors bear responsibility for the integrity and accuracy of the data in the study. LZ: conceived the study and drafted the manuscript. SX, XW, and JC: developed the methods and performed experiments. FM and YC: analyzed the results. JY and ZC: supervised the project. XY and ZH: designed the study and acquired funding.

## Funding

This study was supported in part by grants from the Sichuan Science and Technology Agency Research Foundation (2018JY0164), the National Natural Science Foundation of China (81571114, 81771267, 81701426), the Medical and Health Research Science and Technology Plan Project of Zhejiang Province (2018KY523), and the Public Welfare Science and Technology Plan Project of Wenzhou City (Y20140686, Y20170151).

## Conflict of Interest

The authors declare that the research was conducted in the absence of any commercial or financial relationships that could be construed as a potential conflict of interest.
